# Social Science and the Study of Emerging Infectious Diseases

**DOI:** 10.3201/eid0201.960113

**Published:** 1996

**Authors:** Johannes Sommerfeld, Sandra Lane

**Affiliations:** * Harvard Institute for International Development, Cambridge, Massachusetts, USA † Case Western Reserve University, Cleveland, Ohio, USA


### References

1. Allworth T, O'Sullivan J, Selvey L, Sheridan J. Equine morbillivirus in Queensland. Communicable Diseases Intelligence 1995;19:575.
2. Murray K, Rogers R, Selvey L, Selleck P, Hyatt A, Gould A, et al. A novel morbillivirus pneumonia of horses and its transmission to humans. Emerging Infectious Diseases 1995;1:31-3.

### Social Science and the Study of Emerging Infectious Diseases

Topics related to emerging and reemerging infectious diseases attracted a considerable audience at the annual meeting of the American Anthropological Association, November 15–19, 1995, in Washington, D.C. The meeting had a separate session entitled "Emerging and Reemerging Infectious Diseases: Biocultural and Sociocultural Approaches."

The session brought together anthropologists interested in and working on emerging infectious diseases from various subdisciplinary perspectives. Presentations were made on the following subjects: outline of a research agenda, deforestation and the emergence of infectious diseases in the rain forests of Papua-New Guinea, the cholera epidemic in Latin America, evolutionary aspects of emergent infections, societal impacts of the test for acquired immunodeficiency syndrome, compliance and iatrogenesis in tuberculosis treatment in the United States, patchwork policies that affect long-term treatment of tuberculosis in Nepal and Uganda, the reemergence of schistosomiasis in Egypt, dengue control in Latin America, cultural and political ecologic models of emergent infections, and the politics of leprosy eradication. Abstracts are available from the conference organizers, listed below.

Anthropologists interested in international health and the social science aspects of infectious diseases are organized in a working group called the International Health and Infectious Disease Study Group of the Society of Medical Anthropology (American Anthropological Association). Requests to subscribe to this group's newsletter can be sent to

**Johannes Sommerfeld**
Harvard Institute for International Development
Cambridge, Massachusetts, USA
Phone: 617-495-9791
E-mail: jsommerf@hiid.harvard.edu

**Sandra Lane**
Case Western Reserve University
Cleveland, Ohio, USA
E-mail: sxl45@po.cwru.edu

### WHO Establishes New Rapid-Response Unit for Emerging Infectious Diseases

The World Health Organization (WHO) has established a new rapid-response unit to control and prevent the growing incidence of new and reemerging diseases worldwide. The unit's focus will be improved containment of disease outbreaks, such as that caused by the deadly Ebola virus, which struck Zaire in 1995.

The WHO unit will be called the Division of Emerging Viral and Bacterial Diseases Surveillance and Control (EMC). It will be capable of mobilizing staff from WHO headquarters in Geneva and from the organization's regional offices.

In addition to mobilizing WHO's own technical staff and expertise, EMC will coordinate the activities of the agency's traditional partners, for example, its international network of collaborating centers, bilateral donors, expert advisers, and nongovernmental organizations.

Teams equipped to implement epidemic control measures will be placed on-site within 24 hours' notification of an outbreak. This strategy, when implemented in Zaire, not only rapidly contained the recent Ebola outbreak but also prevented its spread to Kinshasa, the capital city of 2 million.

Among EMC's goals are 1) to strengthen local surveillance and disease control so that countries can develop the early warning systems needed to detect emerging or reemerging diseases through innovative field epidemiology and public health laboratory training programs and 2) to continue WHO's activities in developing a network of public health laboratories to strengthen regional and international collaboration in outbreak detection and control.

EMC will continue to expand WHO's network—termed WHONET—that detects and monitors antibiotic resistance worldwide. WHO will use the information collected to continue to advocate research and development of new antibiotics to replace those that are no longer effective.

Topics related to emerging and reemerging infectious diseases attracted a considerable audience at the annual meeting of the American Anthropological Association, November 15-19, 1995, in Washington, D.C. The meeting had a separate session entitled "Emerging and Reemerging Infectious Diseases: Biocultural and Sociocultural Approaches."

The session brought together anthropologists interested in and working on emerging infectious diseases from various subdisciplinary perspectives. Presentations were made on the following subjects: outline of a research agenda, deforestation and the emergence of infectious diseases in the rain forests of Papua-New Guinea, the cholera epidemic in Latin America, evolutionary aspects of emergent infections, societal impacts of the test for acquired immunodeficiency syndrome, compliance and iatrogenesis in tuberculosis treatment in the United States, patchwork policies that affect long-term treatment of tuberculosis in Nepal and Uganda, the reemergence of schistosomiasis in Egypt, dengue control in Latin America, cultural and political ecologic models of emergent infections, and the politics of leprosy eradication. Abstracts are available from the conference organizers, listed below.

Anthropologists interested in international health and the social science aspects of infectious diseases are organized in a working group called the International Health and Infectious Disease Study Group of the Society of Medical Anthropology (American Anthropological Association). Requests to subscribe to this group's newsletter can be sent to

Johannes Sommerfeld, Harvard Institute for International Development, Cambridge, Massachusetts, USA; Phone: 617-495-9791; E-mail: jsommerf@hiid.harvard.eduSandra Lane, Case Western Reserve University, Cleveland, Ohio, USA; E-mail: sxl45@po.cwru.edu
